# Fecal indicator bacteria and virus removal in stormwater biofilters: Effects of biochar, media saturation, and field conditioning

**DOI:** 10.1371/journal.pone.0222719

**Published:** 2019-09-25

**Authors:** Benjamin P. Kranner, A. R. M. Nabiul Afrooz, Nicole J. M. Fitzgerald, Alexandria B. Boehm

**Affiliations:** 1 Department of Civil and Environmental Engineering, Stanford University, Stanford, California, United States of America; 2 Engineering Research Center (ERC) for Re-inventing the Nation’s Urban Water Infrastructure (ReNUWIt), Stanford, California, United States of America; 3 California State Water Resources Control Board, Sacramento, California, United States of America; 4 Department of Civil and Environmental Engineering, Colorado School of Mines, Golden, Colorado, United States of America; RMIT University, AUSTRALIA

## Abstract

Stormwater biofilters are used to attenuate the flow and volume of runoff and reduce pollutant loading to aquatic systems. However, the capacity of biofilters to remove microbial contaminants remains inadequate. While biochar has demonstrated promise as an amendment to improve microbial removal in laboratory-scale biofilters, it is uncertain if the results are generalizable to the field. To assess biochar performance in a simulated field setting, sand and biochar-amended sand biofilters were periodically dosed with natural stormwater over a 61-week conditioning phase. Impact of media saturation was assessed by maintaining biofilters with and without a saturated zone. Biochar-amended biofilters demonstrated improved *Escherichia coli* removal over sand biofilters during the first 31 weeks of conditioning though media type did not impact *E*. *coli* removal during the last 30 weeks of conditioning. Presence of a saturated zone was not a significant factor influencing *E*. *coli* removal across the entire conditioning phase. Following conditioning, biofilters underwent challenge tests using stormwater spiked with wastewater to assess their capacity to remove wastewater-derived *E*. *coli*, enterococci, and male-specific (F+) coliphage. In challenge tests, biochar-amended biofilters demonstrated enhanced removal of all fecal indicators relative to sand biofilters. Additionally, saturated biofilters demonstrated greater removal of fecal indicators than unsaturated biofilters for both media types. Discrepant conclusions from the conditioning phase and challenge tests may be due to variable influent chemistry, dissimilar transport of *E*. *coli* indigenous to stormwater and those indigenous to wastewater, and differences in *E*. *coli* removal mechanisms between tests. Mobilization tests conducted following challenge tests showed minimal (<2.5%) observable mobilization of fecal indicators, regardless of media type and presence of a saturated zone. While our results emphasize the challenge of translating biochar’s performance from the laboratory to the field, findings of this study inform biofilter design to remove microbial contaminants from urban stormwater.

## Introduction

In response to the threats posed by urban stormwater runoff [1], low impact development (LID) or green infrastructure has emerged as an effective strategy alternative to traditional stormwater management practices [2]. LID seeks to engineer natural systems to mimic the predevelopment hydrology of the urban environment by capturing, infiltrating, evaporating, and detaining runoff to reduce the flux of pollutants from developed areas [3]. Central to LID is the use of distributed stormwater control measures (SCMs) such as biofilters (also referred to as rain gardens, bioinfiltration systems, bioretention systems) to capture and treat stormwater. However, while such systems improve the hydrology of the urban landscape [3], their ability to remove fecal indicator bacteria (FIB) such as *Escherichia coli* and enterococci remains inconsistent and inadequate [4]. Conventional biofiltration media (consisting of sand and compost) has demonstrated limited capacity for FIB removal and some studies have reported greater concentrations of FIB in biofilter effluent than influent [5–7]. To address these inadequacies, biofilters may be amended with alternative geomedia to enhance microbial contaminant removal.

Biochar, a carbonaceous geomedium consisting of pyrolyzed biomass, is attractive as a potential amendment for biofilters owing to its low cost [8], carbon sequestration benefits [9], and sorptive properties [10]. Several laboratory-scale studies demonstrate enhanced *E*. *coli* removal in biochar-amended columns relative to sand-only columns [11–15]. However, the efficacy of biochar amendment depends on various factors, including stormwater chemistry and aging. For example, increased natural organic matter (NOM) concentration in influent stormwater diminishes biochar’s bacterial removal capacity by adsorbing to attachment sites and supporting bacterial growth [16–18]. In addition, physical weathering of biochar has been found to improve its bacterial removal capacity while biological weathering (through biofilm formation) has been found to reduce bacterial removal capacity by changing media surface characteristics [16,18,19]. Under field conditions, significant biofilm formation is expected within biofilter media [19,20]. Additionally, stormwater biofilters in the field are subject to intermittent wetting regimes that impact the observed removal of microbial contaminants. During dry-wet cycles, microbes retained during one wetting event may be mobilized by a subsequent event, thus reducing observed contaminant removal [21]. Furthermore, fluctuations in soil moisture may impact microbial survival and transport in biofilters [22,23]. These issues underscore a need for further investigation of the performance of biochar-amended biofilters under field-like conditions.

This study bridges the gap between laboratory assessment of biochar-amended biofilters and their evaluation in the field using a simulated field setting. We use the term “biofilter” to describe our model system as it is a biologically active filter system (i.e. a system that contains an active microbial community and is not sterilized). As described by Bell et al. [24], nomenclature for LID varies among municipalities and can be inconsistent. While true field assessment of biochar amendment remains desirable, regulatory barriers and environmental risks posed by large-scale experimental biofilters preclude such studies in our local watershed. Alternatively, our study mimics key aspects of the field setting to yield results that are more applicable to the practical application of biochar-amended biofilters. We used natural, unaltered stormwater for the duration of the conditioning phase as opposed to the simplified influent or synthetic stormwater used in previous studies [11–18,25–29]. To our knowledge, there are only two peer-reviewed studies investigating the removal of bacteria from natural stormwater in biochar-amended biofilters [19,30]. Afrooz and Boehm found biochar to enhance the removal of *E*. *coli* and enterococci in biofilters dosed with natural stormwater in a study conducted over 140 days in a laboratory setting [19]. Following a periodic dosing phase of similar duration, Ulrich et al. found that biochar-amendment generally increased removal of *E*. *coli*, though these results were not statistically significant [30]. The conditioning period of the present study exceeds those employed by previous studies, better reflecting the extended timescales relevant to biofilters in the field [31]. Finally, we evaluate the impact of a saturated zone on fecal indicator removal as this design parameter has been found to have inconsistent impacts on indicator removal in previous biofilter studies [19,32,33].

## Materials and methods

### Overview of experiments

**Fig 1:**
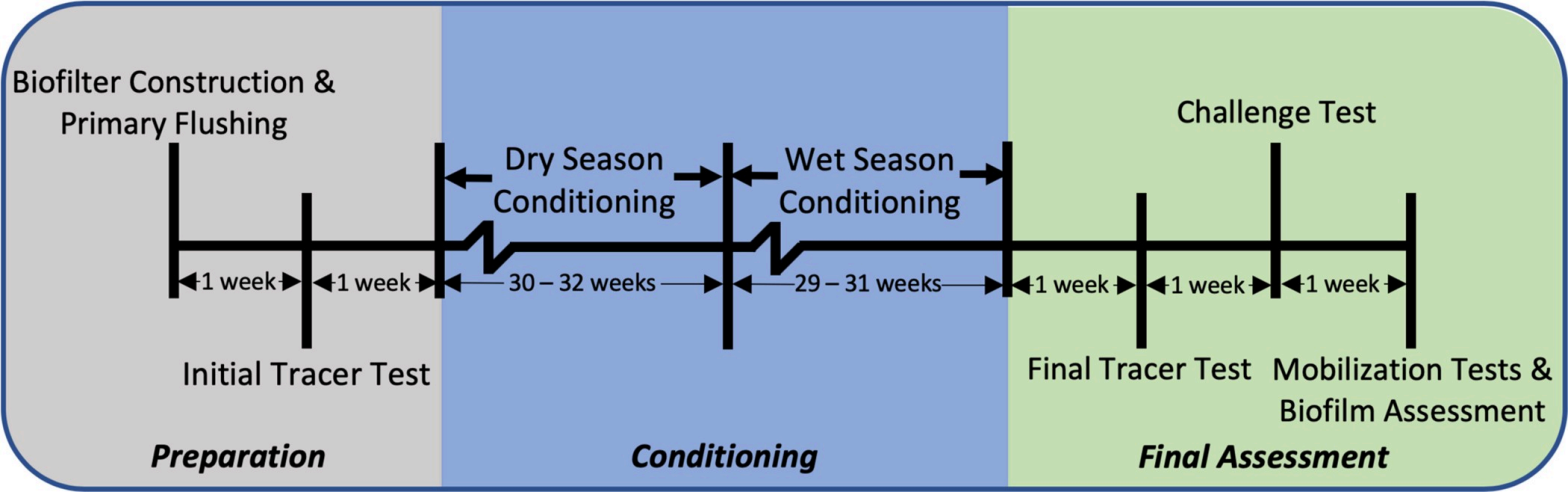
Timeline of the experiment.

Experiments were organized into three phases (Fig 1), as detailed below. The first phase consisted of biofilter construction, flushing to remove fine particulates from biofilter media, and a tracer test to assess hydraulic performance of biofilters. Subsequently, biofilters underwent a 61-week conditioning phase during which all biofilters were periodically dosed with natural stormwater under two different wetting regimes (representing a wet season and a dry season). Following the conditioning phase, a tracer test was conducted to evaluate changes in hydraulic performance. Biofilters were then subject to challenge tests using natural stormwater spiked with 1% raw wastewater (v/v). Finally, biofilters underwent mobilization tests to assess potential mobilization of previously retained fecal indicators. Following mobilization tests, biofilters were deconstructed to analyze biofilm formation on biofilter media.

### Biofilter construction

**Fig 2:**
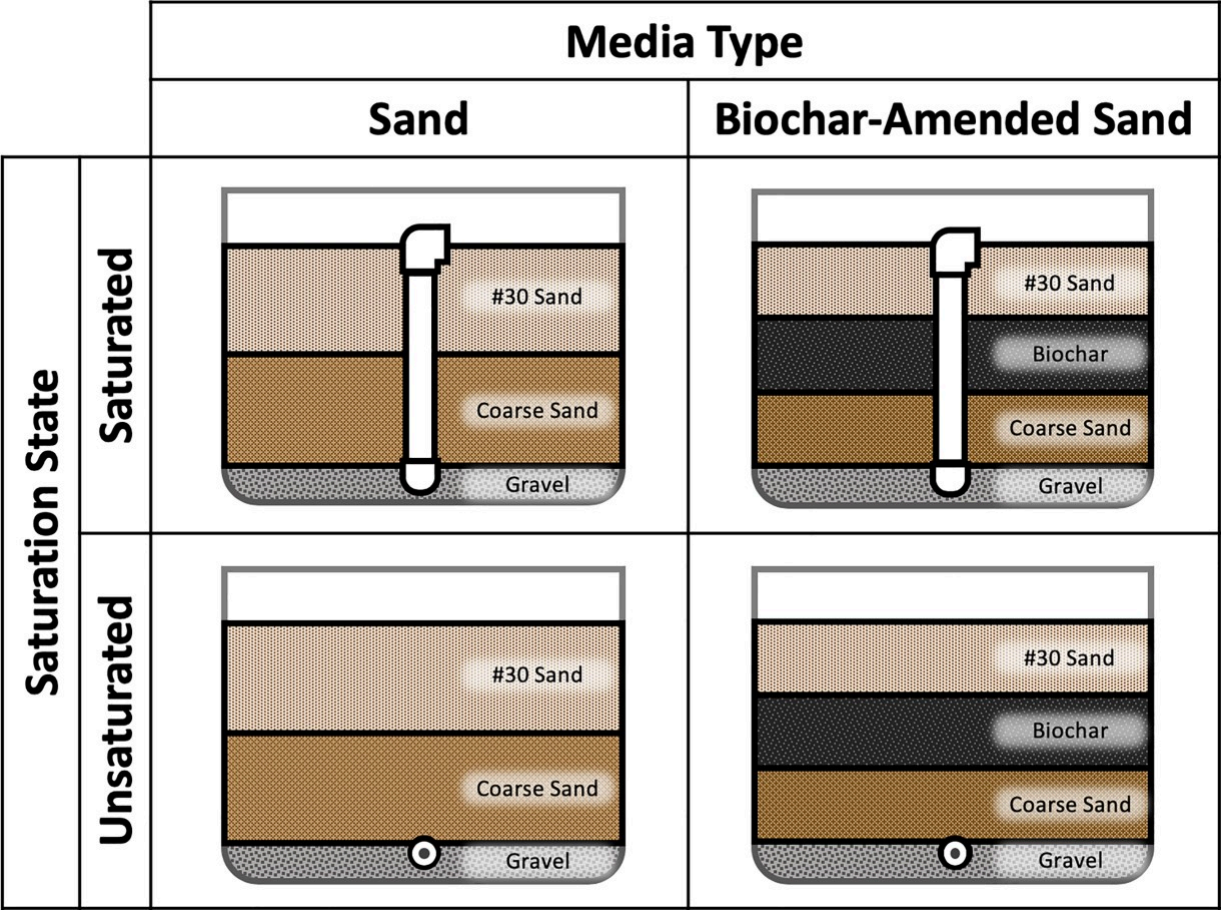
Biofilter experimental conditions. Each condition was tested in triplicate for a total of 12 experimental biofilters.

To assess the influence of media selection and presence of a saturated zone on fecal indicator removal, two different combinations of geomedia, with and without a saturation zone, were used in the experimental biofilters (Fig 2). Each experimental condition was prepared in triplicate, yielding a total of 12 biofilters. Six of the biofilters were packed with sand and biochar layers in a 2:1 volumetric ratio. This ratio was selected based upon current recommendations for organic matter content (20–40%) in biofilter media [34]. As a control, the other six biofilters consisted of exclusively sand. While conventional biofilters generally also contain organic material like compost, the juxtaposition of sand controls with biochar-amended sand filters allowed us to specifically investigate the effect of biochar addition on the performance of biofilters. To investigate the effects of a saturated zone on fecal indicator removal, half of the biofilters of each media type used a standpipe to maintain saturation throughout the entirety of the biofilter media between wetting events. The remaining half of the biofilters were allowed to drain freely via gravity.

Biofilters were constructed by packing media into rectangular, ~41L polypropylene basins measuring approximately 50 cm L x 40 cm W x 30 cm H (IRIS USA, Surprise, AZ). These biofilter dimensions, smaller than typical field-scale biofiltration systems [35,36] but larger than those used in lab-scale studies [37,38], were chosen in part to ensure that the duration of the experiments would be reasonable and could be carried out over a single day. Although the dimensions of the biofilters do not precisely mimic those of field scale systems, the particle sizes of the geomedia are comparable with the local biofiltration soil media specification [34]. Moreover, identical sizing of control (sand) and test (biochar-amended sand) biofilters allowed us to compare the effect of biochar amendment on the performance of biofilters under field conditions. The outlet of each biofilter consisted of 1.3 cm diameter polyvinyl chloride (PVC) pipe running lengthwise along the bottom of the biofilter. To allow water to drain and prevent clogging, holes were drilled along the pipe’s length and the pipe was screened with filter sock fabric (Carriff Engineered Fabrics Corporation, Midland, NC). All biofilters incorporated a 2.5 cm layer of gravel (¼”-½” pea pebbles; Vigoro Corporation, Chicago, IL) at the bottom to cover the outlet pipe and further prevent clogging. Prior to packing, gravel was rinsed thoroughly with tap water to remove fine particulate matter.

Biochar was sourced from the Sonoma Ecology Center, Eldridge, CA. According to manufacturer specifications, the biochar feedstock consisted of 60% Monterey Pine, 20% Eucalyptus, 10% Bay Laurel, and 10% mixed hardwood and softwood. Pyrolysis temperature peaked at 394°C (742°F): details of the pyrolysis are provided in S1 Table. Prior to packing into biofilters, biochar was crushed and passed through a non-standard sieve comparable in sieve opening to a #30 sieve (nominal sieve opening = 595 μm).

Following installation of an outlet pipe and gravel layer in each basin, porous media was dry packed in layers. Dry packing was selected over wet packing due to the tendency of biochar to float during wet packing [17]. In biochar-amended biofilters, the first layer consisted of 7.6 cm of coarse sand (Lapis Lustre F-108; effective size 0.70–0.80 mm) (CEMEX, Monterey, CA). This was then overlaid with a 7.6 cm layer of biochar and a 7.6 cm layer of #30 Lapis Lustre sand (effective size 0.21–0.31 mm) (CEMEX, Monterey, CA). Rather than blending biochar with sand, a discrete layer of biochar sandwiched between sand layers was used. This prevented leaching of fine biochar particles from each biofilter and represented a simplified construction technique that may be preferred in true field biofilters. Sand biofilters were similarly packed, though the biochar layer was omitted, and each sand layer’s depth was increased to 11.4 cm. Following packing, each biofilter basin had approximately 4.5 cm of freeboard remaining to accommodate a ponding zone.

Following construction, biofilters were flushed with 20 pore volumes of tap water to remove fine particulate matter. As gravimetric determination of the pore volume of the biofilters was impractical, the pore volume (PV) of each biofilter was assumed to be 13.3 L, equivalent to 40% of the estimated total porous media volume. This reflects the porosity measured previously in smaller biofilters packed in the same manner and containing the same media [19,26,39].

Biofilters were sheltered in a tent at the Codiga Resource Recovery Center on the Stanford University campus. Thus, biofilters were exposed to ambient temperatures (ranging from ~5°C to ~35°C) but were sheltered from precipitation and sunlight over the course of conditioning and final assessment. Biofilters were operated in a gravity flow configuration to mimic the conditions experienced by stormwater biofilters in the field.

### Tracer test

Biofilters were subject to tracer tests to evaluate their hydraulic performance prior to the conditioning phase. For distributing tracer test influent evenly over the biofilters, three parallel, capped soaker hoses (Swan Products, Marion, OH) were installed lengthwise atop each biofilter. To secure hoses, three plastic stakes (measuring 13 cm in length) were inserted into the biofilter media. Given that this likely impacts the hydraulic performance, these stakes were left in place through the duration of the study to ensure that biofilter hydraulic conditions might reflect those assessed in the tracer test.

Tracer test influent was prepared by adding 10 mM stock NaBr solution (Sigma-Aldrich, St. Louis, MO) to tap water to a final concentration of 2 mM NaBr. Prior to beginning the tracer test, biofilters were presaturated with tap water. A peristaltic pump (Masterflex L/S, Cole-Parmer, Vernon Hills, IL) was used to deliver influent to the soaker hoses at a rate of 0.37 L/min (equivalent to 12.7 cm/hr or 5 in/hr across the biofilter surface). When effluent began flowing from each biofilter outlet, duplicate effluent samples were immediately collected in sterile glass test tubes and Br^-^ concentrations were assessed using a HI4102 bromide ion selective electrode (Hanna Instruments, Woonsocket, RI). Subsequent effluent samples were collected in duplicate every 18 minutes (reflective of 0.5 pore volumes at the aforementioned influent flow rate).

Effluent bromide concentrations were normalized to influent bromide concentrations for plotting. To address normalized effluent concentrations that exceeded 1 (which was found in a small proportion of effluent samples), effluent concentrations were normalized to the maximum effluent bromide concentration observed for subsequent analysis. The FORTRAN program *trafit1d* from Seagren et al. [40] was adapted for MATLAB (MathWorks, Natick, MA) to fit the one-dimensional, non-reactive solute transport model of Parker and van Genuchten [41] to the experimental tracer data. This analysis yielded best-fit values for the hydrodynamic dispersion coefficient and pore water velocity.

### Conditioning phase

During the conditioning phase, biofilters were periodically dosed with natural stormwater over 61 weeks. Between March and October 2017, biofilters were dosed once every two weeks (mean antecedent dry days = 14.0 d; σ = 1.5 d). This represents a relatively dry regime mimicking wetting that may occur during rare irrigation events in the dry season of a Mediterranean climate (such as that typical to California). During this dry regime, influent was sampled during each conditioning event and biofilter effluent samples were collected once every four weeks. Between October 2017 and May 2018, biofilters were dosed once per week (mean antecedent dry days = 7.0 d; σ = 1.2 d). This reflects a relatively wet regime to mimic the wet season of a Mediterranean climate. During the wet regime, influent was sampled during each conditioning event and effluent was sampled once every two weeks.

During each conditioning event, stormwater from a nearby creek was collected in clean, 121 L plastic basins prior to its immediate application to the biofilters. Given that water was available in the creek on a consistent basis, this influent stormwater reflects both dry and wet weather runoff. Although soaker hoses (which were installed for the initial tracer test) were considered for distributing influent flow over each biofilter, their use was abandoned due to concerns regarding clogging of the hoses by sediment in stormwater. Instead, clean plastic watering cans were used to distribute stormwater over each biofilter. During each conditioning event, 28.4 liters (~2.1 pore volumes) of stormwater were applied to each biofilter. This volume was selected as to ensure complete flushing of the biofilters during each conditioning event with a factor of safety of 2. Over the entire conditioning phase, each biofilter was dosed with 100 PV of stormwater.

In the event that a biofilter’s ponding zone reached capacity before the entire influent volume was applied, the ponding zone was allowed to partially drain prior to addition of the remaining influent to prevent water from spilling from the biofilter. Care was taken to maintain continuous influent flow. After each biofilter ceased draining, a 250 mL aliquot of the composite effluent was collected for enumeration of *E*. *coli*.

### Challenge test

Approximately one week after the conditioning phase ended (62^nd^ week), biofilters were subject to a second tracer test to assess potential changes in their hydraulic performance. This tracer test mimicked the initial tracer test with regards to flow rate, flow distribution with soaker hoses, and sampling regime. However, final tracer test influent consisted of natural stormwater spiked with NaBr (rather than tap water spiked with NaBr). This modification was made to avoid disturbing biofilm that may have accumulated in the biofilters during the conditioning phase. The final tracer test was conducted without pre-saturating the biofilters, allowing for only qualitative comparisons with the initial tracer test results.

Approximately 4 days (Mean antecedent dry days = 3.9 d, σ = 1.0 d) after the second tracer tests, biofilters were subject to a challenge test to assess their capacity to removal *E*. *coli*, enterococci, and F+ coliphage from stormwater. To select the influent volume for the challenge test, the Soil Conservation Service (SCS) runoff curve method [42] was used to estimate the runoff volume generated by a 1-hour storm with a 25-year recurrence interval in the local water watershed (Matadero Creek, CA). This represents a relatively large storm event and yielded an influent volume approximately twice that of the volume used during each conditioning event. Land use information for the watershed was collected from the Santa Clara Valley Urban Runoff Pollution Prevention Program [43] and soil group data was collected from the USDA Natural Resources Conservation Service [44]. For estimating runoff volume imposed on stormwater control measures (SCM) from the given storm, it was assumed that SCMs cover 1% of the overall watershed area (on par with typical infiltration basin sizing for a drainage area) [45]. These calculations yielded an influent volume of approximately 56 L for each biofilter for the challenge test.

Influent in the challenge test consisted of natural stormwater (collected from the same creek used during the conditioning phase) spiked with 1% raw wastewater (v/v). Raw wastewater was collected from the Codiga Resource Recovery Center at Stanford University, a facility for testing pilot scale wastewater treatment systems that processes 150 m^3^/day of wastewater from the residential part of the campus and several student dining halls. Similar to the conditioning phase, influent was applied to the surface of each biofilter using clean plastic watering cans.

Challenge test effluent samples were collected as sequential, 0.5 pore volume fractions in sterile plastic basins. In total, eight effluent fractions were collected from each biofilter, reflecting 0.5 pore volume increments up to 4.0 pore volumes. Prior to sample collection, plastic basins were sterilized with 70% ethanol solution and dried. Aliquots for each effluent fraction were transferred from plastic basins into autoclaved polypropylene bottles and stored on ice for later analysis. Samples were processed within 6 hours of collection.

### Mobilization test

Approximately one week after challenge tests, tests were conducted to assess the potential mobilization of previously retained fecal indicators. Mobilization test influent was prepared by collecting natural stormwater from the creek at the field site in sterile carboys. This stormwater was subsequently autoclaved at 121°C for 45 minutes before one pore volume (13.3 L) was applied to duplicate biofilters for each experimental condition. One biofilter of each condition was omitted from the mobilization test due to sample processing limitations (i.e. 2 of the 3 replicates from each treatment were used in the mobilization tests). Autoclaved stormwater was applied using watering cans sterilized with 70% ethanol solution and dried. Sequential effluent fractions were collected at increments of 0–2 L, 2–5 L, 5–9 L, and >9 L (i.e. from 9 liters of effluent until the biofilter ceased draining freely) for enumeration of fecal indicators. A volume-weighted average of fecal indicator concentrations was used to calculate a composite effluent sample for subsequent analyses.

### Fecal indicator enumeration

*E*. *coli* concentrations were measured in conditioning phase influent and effluent samples using a Most Probable Number (MPN) assay. Duplicate 1:10 dilutions of each sample were prepared by pipetting 10 mL aliquots into 90 mL bottles of Butterfield’s buffer (Maine Manufacturing, Sanford, ME). Colilert-18 (IDEXX, Westbrook, ME) and the Quanti-Tray 2000 system (IDEXX, Westbrook, ME) were used to determine *E*. *coli* concentration. Following incubation and positive well counts, concentrations of *E*. *coli* were estimated using the method described by Jarvis et al. [46]. Duplicates of the 97-well Quanti-Tray 2000 were treated as a single tray of 194 wells (98 large wells and 96 small wells) for the purposes of determining MPN and uncertainty estimates. Similar methods were employed to enumerate enterococci for samples collected during the challenge and mobilization tests. For enumeration of enterococci, Enterolert test kits (IDEXX, Westbrook, ME) were used with the Quanti-Tray 2000 system. For each challenge test sample, ten-fold and thousand-fold dilutions were prepared. Similarly, mobilization test samples were assessed using a thousand-fold dilution and a non-dilute sample.

To preserve coliphage for subsequent enumeration, membrane filtration was used to concentrate phage from challenge and mobilization test samples after addition of MgCl_2_ following the methods of Sinton et al. [47]. Male-specific coliphage were enumerated using an elution and double agar layer method adapted from Sinton et al. [47] and Mendez et al. [48] using host F_amp_ *E*. *coli* (ATCC 700891, American Type Culture Collection, Manassas, VA). Positive and negative controls were prepared using raw wastewater and sterile, deionized water, respectively.

### Biofilm characterization

Immediately following mobilization tests, biofilters were deconstructed and media was collected to characterize biofilm formation. Duplicate samples were collected from two biofilters from each experimental condition (specifically, the biofilters that were previously subject to mobilization tests). Media samples were collected at three depths within each biofilter at the midpoint of each media layer. In sand biofilters where only two unique media layers were present, the intermediate depth sample was collected at the interface of the two sand layers. Samples were collected with a steel garden trowel (sterilized with 70% ethanol and rinsed with autoclaved, deionized water) and stored in sterile, polystyrene petri dishes. Samples were transported on ice to the laboratory and stored at 4°C for no more than 4 hours before biofilm characterization. Biofilm characterization was performed following an adenosine triphosphate (ATP) based biomass quantification method adapted from Velten et al. [49] and used previously by Afrooz and Boehm [18].

### Data analysis

Log removal values were calculated for each effluent sample collected during the conditioning phase and challenge tests using Eq 1:

$$Log\;Removal=-\log_{10}\left(\frac{C}{C_{0}}\right)$$ (1)

where, C = effluent fecal indicator concentration (MPN/100 mL or PFU/100 mL) and C_0_ = influent fecal indicator concentration (MPN/100 mL or PFU/100 mL).

To evaluate uncertainty in log removal values for *E*. *coli* and enterococci, we employed a Monte Carlo simulation to account for uncertainties in influent and effluent concentrations. Each concentration represents a maximum likelihood estimator $\hat{\mu }$, the value at which a likelihood function is maximized. ln $\hat{\mu }$ follows an approximately normal distribution with variance ${\hat{\sigma }}_{\ln\hat{\mu }}^{2}$. Equations for calculating $\hat{\mu }$ and ${\hat{\sigma }}_{\ln\hat{\mu }}^{2}$ using the parameters and results of a microbial density assay are outlined by Jarvis et al. [46]. By calculating $\hat{\mu }$ and ${\hat{\sigma }}_{\ln\hat{\mu }}^{2}$ for each sample, a normal distribution was defined from which to draw concentration values. Influent and effluent concentration values were used to calculate simulated log removal values. For each effluent sample, 10,000 log removal values were simulated. Simulation results from triplicate biofilters were aggregated and the median and interquartile range of log removal values were determined.

To evaluate the association of media type, saturated zone, biofilter age, and influent *E*. *coli* concentrations upon *E*. *coli* removal during the conditioning phase, we used multiple linear regression:

$$Log\;removal=\beta_{0}+\beta_{1}(Media\;Type)+\beta_{2}(Saturated\;Zone)+\beta_{3}(Age)+\beta_{4}({log}_{10}(\frac{C_{0,event}}{C_{0,GM}})$$ (2)

“Media type” is a binary variable, with 1 indicating biochar-amended biofilters and 0 indicating sand biofilters. “Saturated zone” is similarly a binary variable, with 1 indicating fully saturated media and 0 indicating unsaturated media. “Age” is the number of weeks that had elapsed since the beginning of the conditioning phase (rounded to the nearest integer). The last variable on the right-hand side of Eq 2 represents the log_10_-transformed ratio of influent *E*. *coli* concentration for a given sampling event (C_0,event_) to the geometric mean influent *E*. *coli* concentration for all sampling events included in the model (C_0,GM_)_._ The model intercept (β_0_) thus represents the expected *E*. *coli* log removal for an unaged, unsaturated, sand biofilter when influent *E*. *coli* concentration is the geometric mean of influent *E*. *coli* concentrations.

When biofilters of the same media type and saturation state shared influent distributed from the same stormwater sample (i.e. a single estimate of influent *E*. *coli* concentration), effluent *E*. *coli* concentrations were averaged across experimental replicates for modeling. This averaging prevented pseudo-replication that would result if a single estimate of influent *E*. *coli* concentration was paired to unique estimates of effluent *E*. *coli* concentration. *E*. *coli* concentrations outside of detection limits were omitted from the regression. Linear regression was performed using least-squares fitting in MATLAB.

To evaluate biofilter performance during the challenge tests, breakthrough curves were prepared for each fecal indicator. In instances where effluent F+ coliphage concentration fell below the lower limit of quantification (LLOQ) (1 PFU/100 mL), the concentration was set at half the LLOQ for the purposes of calculating log removal values. When influent coliphage concentration exceeded the upper limit of quantification (ULOQ) (1000 PFU/100 mL; ~17% of influent samples), the influent concentration was set as the mean coliphage concentration for biofilters that underwent challenge tests on the same date. A single replicate of a biochar-amended, saturated biofilter was omitted from breakthrough curve calculations due to its aberrant behavior as it exhibited steadily decreasing phage concentrations in consecutive effluent fractions (as compared to the increasing concentrations observed across all other biofilters). Additionally, the first three effluent fractions collected from this biofilter suggested leeching of coliphage, with greater concentrations in effluent than influent.

The percent of fecal indicators mobilized during the mobilization test was calculated as follows:

$${\left(Percent\;Mobilized\right)}_{i}=100*\frac{N_{i,total\;mobilized}}{\left(N_{i,total\;challenge\;influent}-N_{i,total\;challenge\;effluent}\right)}$$ (3)

*(Percent Mobilized)*_*i*_ represents the percent of indicator *i* mobilized during the mobilization test. *N*_*i*,*total mobilized*_ is the total number of indicator *i* mobilized from a biofilter during the mobilization test. *N*_*i*,*total challenge influent*_ and *N*_*i*,*total challenge effluent*_ represent the total number of indicator *i* in the influent and effluent of each biofilter’s challenge test, respectively. *N*_*i*,*total mobilized*_ and *N*_*i*,*total challenge effluent*_ were each calculated by multiplying the concentration of *i* in each effluent fraction by each fraction’s respective volume. *N*_*i*,*total mobilized*_ and the total effluent volume for the mobilization test were used to estimate the concentration of *i* in a calculated composite effluent sample. The total effluent volume was equivalent to the total influent volume less 0.2 PV, which was the volume of the influent retained in the biofilters during each wetting event (as determined during previous wetting events over the conditioning and challenge phases of the experiments). While the volume retained within the biofilters reflects an estimate based upon field observations, it should be noted that modification to this estimate does not notably impact the mobilization test conclusions (as the number of retained indicators is approximately 2–3 orders of magnitude greater than the number mobilized; see Results). For mobilization analyses, the concentration of indicators contained in the sterilized influent stormwater was ignored. These concentrations fell below detection limits in most cases and were orders of magnitude smaller than the fecal indicator concentrations observed in mobilization test effluent.

Uncertainty in the analyses of *E*. *coli* and enterococci mobilization was estimated through a Monte Carlo simulation comparable to that described previously. MPN estimates for mobilization test influent and effluent samples were simulated and used to compute values for input into Eq 3. The percent of each indicator bacteria mobilized was simulated 10,000 times for each biofilter. Results from duplicate tests for each experimental condition were aggregated and median and interquartile ranges were determined.

## Results

### Tracer tests

**Fig 3:**
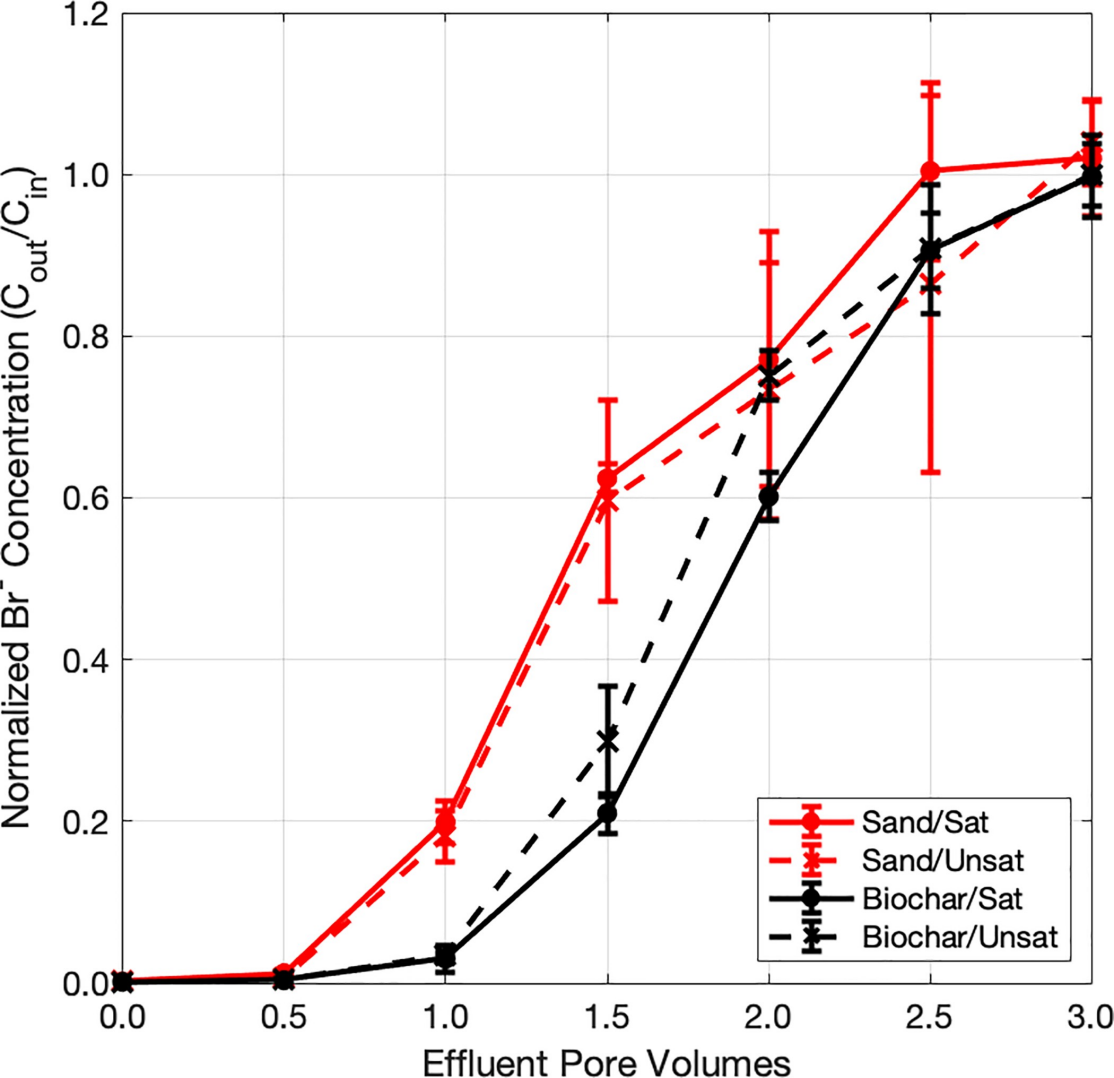
Br^-^ breakthrough curves from initial tracer test. Each point represents the average normalized Br^-^ concentration from duplicate technical replicates and triplicate biological replicates for each experimental condition. Error bars represent 1 standard deviation.

Breakthrough curves from the initial tracer test are depicted in Fig 3. Visual inspection of breakthrough curves indicated that sand biofilters demonstrated earlier breakthrough than biochar-amended biofilters. This observation was corroborated by the best-fit pore water velocities, which were generally greater in sand biofilters (25.4 ± 2.4 cm/hr and 22.4 ± 1.6 cm/hr for saturated and unsaturated biofilters, respectively) than in biochar-amended biofilters (20.0 ± 0.5 cm/hr and 21.7 ± 0.7 cm/hr for saturated and unsaturated biofilters, respectively). Hydrodynamic dispersion coefficients were similarly greater in sand biofilters (45.0 ± 12.0 cm^2^/hr and 66.9 ± 19.0 cm^2^/hr for saturated and unsaturated biofilters, respectively) than in biochar-amended biofilters (14.0 ± 1.2 cm^2^/hr and 17.0 ± 7.3 cm^2^/hr for saturated and unsaturated biofilters, respectively).

Biofilters were not pre-saturated for the final tracer test (breakthrough curves for which are depicted in S2 Fig), limiting comparison with the initial tracer test as well as comparison of saturated and unsaturated biofilters. However, qualitative evaluation of the final tracer test results indicated that sand and biochar-amended biofilters exhibited similar hydraulic characteristics, regardless of the presence a saturated zone. Tracer breakthrough of saturated sand and biochar-amended biofilters in the final tracer tests was ostensibly similar to that observed for all sand biofilters during the initial tracer tests. Thus, it appears that conditioning resulted in slightly shorter hydraulic residence times for biochar-amended biofilters but did not notably modify hydraulic performance of sand biofilters. This suggests that clogging did not appreciably impact hydraulic performance across all biofilters.

### Conditioning phase *E*. *coli* removal

Concentrations of *E*. *coli* in natural stormwater applied to biofilters ranged from 20 MPN/100 mL to greater than the upper limit of quantification (ULOQ; 28,527 MPN/100 mL) during the conditioning phase. These concentrations are within the range of *E*. *coli* reported for stormwater in a systematic review [50]. Influent *E*. *coli* concentrations above the ULOQ (~2% of samples) were omitted from subsequent analyses. The geometric mean influent *E*. *coli* concentration for all conditioning events (regardless of whether effluent was sampled) was 785 MPN/100 mL. The geometric mean influent *E*. *coli* concentration used in the linear regression model (C_0,GM_ in Eq 2) was 811 MPN/100 mL, representing the geometric mean of influent *E*. *coli* concentrations coupled to effluent samples.

Conditioning phase influent *E*. *coli* concentrations within each experimental condition were found to be non-normally distributed (Shapiro-Wilk test, *p* > 0.05). No significant differences were found between *E*. *coli* influent concentrations for each experimental condition (Wilcoxon rank-sum test, *p* > 0.05) which is consistent with the experimental method which involved applying the same influent (from a large tank) to each biofilter.

Concentrations of *E*. *coli* in conditioning phase biofilter effluent, regardless of experimental configuration, ranged from below the LLOQ (5 MPN/100mL; ~1% of samples) to greater than the ULOQ (28,527 MPN/100 mL; ~1% of samples). Samples with effluent *E*. *coli* concentrations outside the limits of quantification were omitted from subsequent analyses. The geometric mean effluent *E*. *coli* concentration across all experimental configurations was 275 MPN/100 mL. Geometric mean effluent *E*. *coli* concentrations for each experimental configuration are reported in S2 Table in the Supplementary Information.

**Fig 4:**
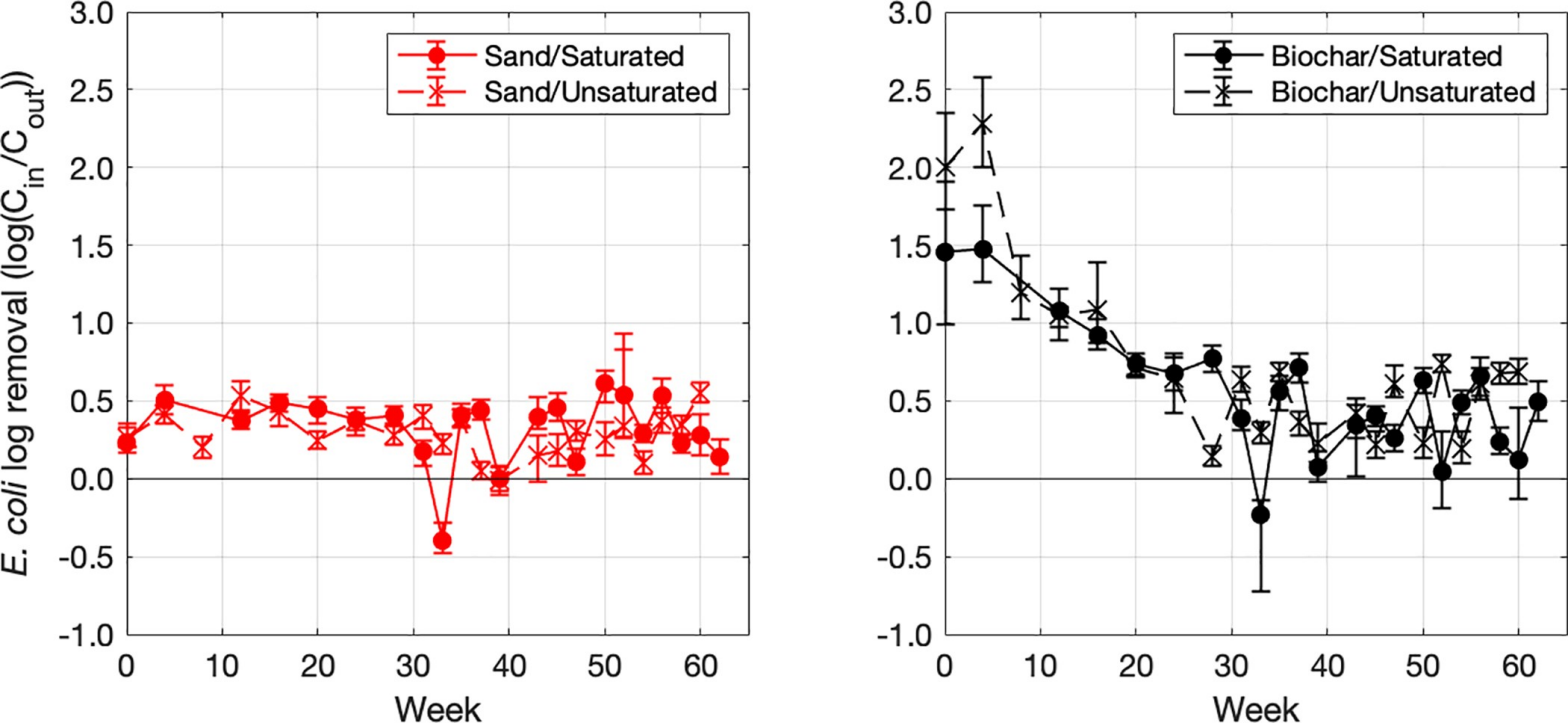
Log_10_ removal of E. coli during the >60 week conditioning phase. Each point represents the median log_10_ removal value yielded by the Monte Carlo simulation (described in the main text) while error bars represent the interquartile range of log_10_ removals. Recall that E. coli were not seeded into the stormwater used for conditioning and hence represent indigenous stormwater E. coli. The results from the sand-only biofilters are shown in the left panel and results for the biochar-amended sand biofilters are shown on the right.

Log removal values for *E*. *coli* during the conditioning phase ranged from -0.78 to 2.41 across all biofilters and all time points (Fig 4). In approximately 94% of sampling events, influent *E*. *coli* concentrations exceeded effluent concentrations, indicating that biofilters largely removed *E*. *coli* from influent stormwater. In the most extreme leaching cases (log removal < -0.2), we observed that influent *E*. *coli* concentration in the preceding conditioning event was elevated (~95^th^ percentile of influent *E*. *coli* concentrations) while influent *E*. *coli* concentration during the leaching event was relatively low (~30^th^ percentile of influent *E*. *coli* concentrations).

Linear regression analysis indicated that media type and biofilter age were clearly associated with *E*. *coli* log_10_ removal during the conditioning phase (*p* < 0.05, Table 1), with log removals greater in biochar-amended biofilters and diminishing with age. Saturated zone was not significantly associated with *E*. *coli* removal (*p* = 0.60), although removal was diminished when a saturated zone was present (coefficient < 0). Higher influent *E*. *coli* concentration resulted in higher log_10_ removals, although the *p*-value did not reach our cut off value for significance (p = 0.11).

**Table 1:** Multiple linear regression model results for conditioning phase.

Variable	Coefficient	Standard error	*t*-statistic	*p*-value
Intercept	0.711	0.094	7.573	p < 10^−5^
Age	-0.011	0.002	-5.559	p < 10^−5^
Media Type	0.380	0.075	5.029	p < 10^−5^
Saturation Zone	-0.040	0.076	-0.530	0.60
${log}_{10}(\frac{C_{0,event}}{C_{0,GM}})$	0.106	0.066	1.616	0.11
n = 94; RMSE = 0.366, R^2^ = 0.41; p-value = 1.2 × 10^−9^				

To investigate temporal changes in the model parameters during the conditioning phase, additional linear regressions were prepared to determine the age at which biofilter media type was no longer a significant predictor of *E*. *coli* removal. Successive models indicated that media type ceased being a significant predictor of *E*. *coli* log removal after week 31 of the conditioning phase (*p* > 0.05).

### Challenge test

The concentration of *E*. *coli* in the challenge test influent ranged from 2.73 × 10^4^ MPN/100 mL to 2.72 × 10^5^ MPN/100 mL, with a geometric mean of 1.38 × 10^5^ MPN/100 mL. Though these concentrations are elevated relative to those observed during the conditioning phase, they are comparable with the maximum *E*. *coli* concentrations observed in natural stormwater [51]. The concentration of enterococci in challenge test influent ranged from 1.69 × 10^4^ MPN/100 mL to 4.60 × 10^4^ MPN/100 mL, with a geometric mean of 2.90 × 10^4^ MPN/100 mL. Finally, F+ coliphage concentrations in challenge test influent ranged from 4.2 × 10^1^ PFU/100 mL to greater than the ULOQ (1000 PFU/100 mL; ~17% of influent samples). The geometric mean influent F+ coliphage concentration was 1.8 × 10^2^ PFU/100 mL.

**Fig 5:**
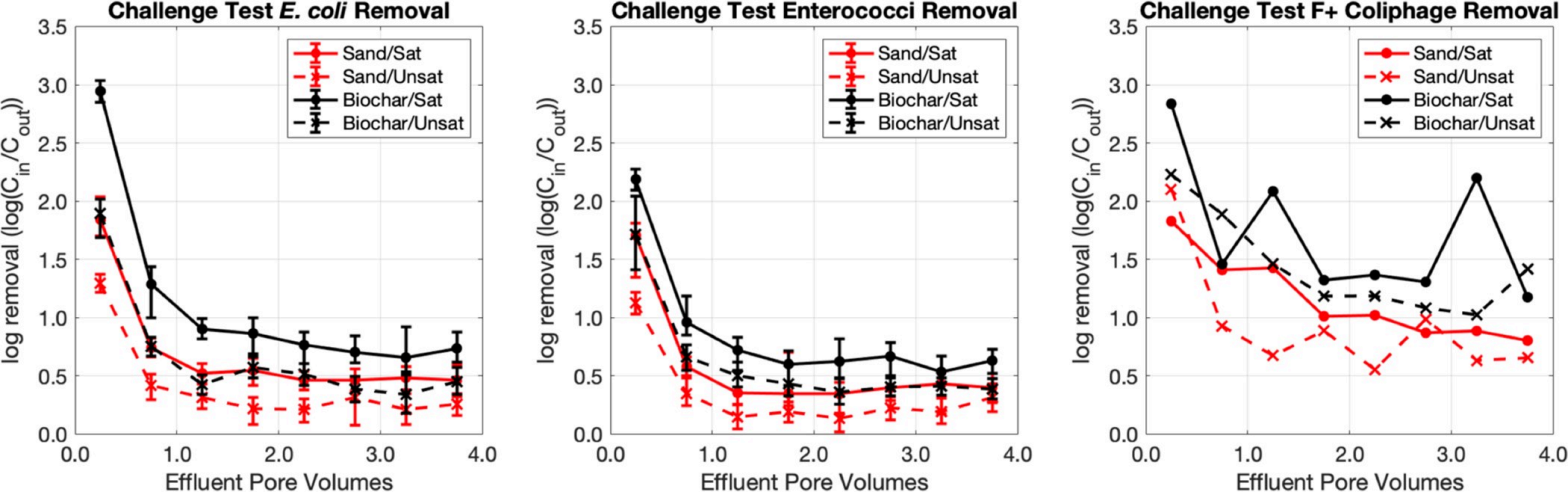
Log_10_ removals of fecal indicators in challenge test effluent fractions. The horizontal position of each point is fixed at the midpoint of the effluent fraction that it represents (e.g. the data for the 0.0–0.5 effluent fraction is fixed at 0.25 effluent pore volumes). For E. coli and enterococci plots, each point represents the median log_10_-removal value yielded by the Monte Carlo simulation while error bars represent the interquartile range of log_10_-removals.

Breakthrough curves, plotted as log removal of *E*. *coli*, enterococci, and F+ coliphage, from the challenge tests are depicted in Fig 5. While *E*. *coli* and enterococci curves reach a plateau following discharge ~1.0 PV of effluent, F+ coliphage breakthrough curves do not demonstrate a clear plateau (though log-removal values for coliphage somewhat stabilize following discharge of ~1.5 PV). Overall, coliphage concentrations in effluent fractions were more variable than FIB concentrations. This was particularly notable for the saturated, biochar-amended biofilters.

The average log_10_ removal values for each fecal indicator, calculated using the log_10_ removals from effluent fractions after 1.0 PV for *E*. *coli* and enterococci and after 1.5 PV for coliphage, are reported in Table 2. On average, log_10_ removal values for all fecal indicators were higher for biochar-amended biofilters than for sand biofilters. Similarly, when controlling for geomedia type, saturated biofilters demonstrated greater average log removal than unsaturated biofilters for all fecal indicators assessed. Across all biofilter configurations, average log_10_ removal of *E*. *coli* was slightly greater than that of enterococci, though biofilters were most effective at removing F+ coliphage.

**Table 2:** Fecal indicator log_10_ removal during challenge tests. Errors represent standard deviations of log_10_ removal values.

	Experimental Configuration			
	Sand/Sat	Sand/Unsat	Biochar/Sat	Biochar/Unsat
*E*. *coli*	0.49 ± 0.10	0.25 ± 0.15	0.78 ± 0.17	0.45 ± 0.14
Enterococci	0.39 ± 0.17	0.20 ± 0.12	0.62 ± 0.16	0.43 ± 0.11
F+ coliphage	0.91 ± 0.25	0.76 ± 0.40	1.47 ± 0.54	1.15 ± 0.29

### Mobilization test

In all mobilization test influent samples, *E*. *coli* and F+ coliphage concentrations were below their respective LLOQ (1 MPN/100 mL and 1 PFU/100 mL, respectively). Enterococci concentrations were below the LLOQ (1 MPN/100 mL) in ~83% of mobilization influent samples and were 1 MPN/100 mL in the remaining samples. Across composite effluent samples, *E*. *coli* concentrations ranged from 291 MPN/100 mL to 4089 MPN/100 mL with a geometric mean of 1053 MPN/100 mL. Effluent enterococci concentrations ranged from 146 MPN/100 mL to 720 MPN/100 mL with a geometric mean of 287 MPN MPN/100 mL. Effluent F+ coliphage concentrations ranged from below the LLOQ (0.01 PFU/100 mL) to 2.2 PFU/100 mL, with an arithmetic mean of 0.7 PFU/100 mL.

**Fig 6:**
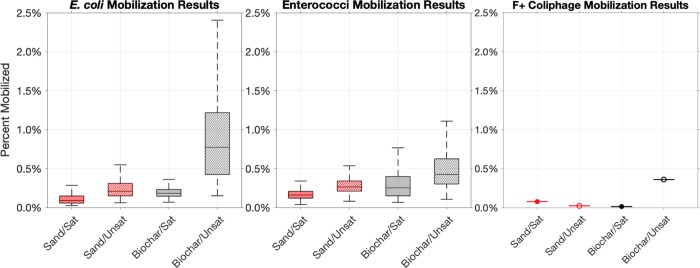
Fecal indicator mobilization results. Boxplots of *E*. *coli* and enterococci depict the results of Monte Carlo simulations. Boxes depict the interquartile range of simulation results, the line through the center of the box represents the median and whiskers extend to ±2.7σ. Results from Monte Carlo simulations were comparable to deterministic estimates of fecal indicator mobilization. Plot of F+ coliphage depicts the average coliphage mobilization measured for each experimental condition.

Mobilization of previously retained fecal indicators was minimal across all four experimental conditions according to Eq 3, which considers the percent of retained *E*. *coli* mobilized (Fig 6). *E*. *coli* mobilization ranged from 0.05% to 1.2% regardless of experimental configuration. Biochar-amended biofilters exhibited greater *E*. *coli* mobilization (0.5%) than sand biofilters (0.2%). Similar results were observed for enterococci mobilization, with biochar-amended filters demonstrating 0.4% mobilization and sand biofilters exhibiting 0.2% mobilization. Unsaturated biofilters exhibited greater *E*. *coli* mobilization (0.5%) than sand biofilters (0.1%). Media had a similar impact upon enterococci mobilization, with unsaturated biofilters exhibiting 0.4% mobilization and saturated biofilters exhibiting 0.2% mobilization. The concentrations of *E*. *coli* in the composite effluent during the mobilization tests ranged from 2.91 × 10^2^ MPN/100 mL to 4.09 × 10^3^ MPN/100 mL. Concentrations of enterococci in composite mobilization test effluent ranged from 1.46 × 10^2^ MPN/100 mL to 7.20 × 10^2^ MPN/100 mL.

In three of the eight biofilters that underwent mobilization tests, no mobilization of F+ coliphage was observed. When coliphage mobilization was observed, it ranged from 0.006% to 0.7% with an average of 0.1%. Coliphage mobilization was less than 0.2% across all experimental conditions, although 0.7% mobilization was observed in one of the unsaturated, biochar-amended biofilters.

### Biofilm assessment

ATP density (a proxy for active biomass) on biofilter media varied greatly within biofilters (Table 3). Across sand biofilters, the average ATP density was three times higher in the upper layer of sand (8.6 ± 3.6 ng ATP/g dry collector) relative to samples taken from the middle and bottom portions of the biofilters (2.9 ± 1.9 ng ATP/g dry collector and 2.7 ± 0.6 ng ATP/g dry collector, respectively). This pattern was not observed in biochar-amended biofilters as average ATP density was much greater (approximately 55 and 70 times higher than top and lower layer, respectively) within the middle, biochar layer for both saturated and unsaturated biofilters (Table 3). Regardless of the saturation conditions, ATP densities in the biochar-containing middle layer were significantly higher than middle, sand-only layers: 50 and 120 times higher for saturated and unsaturated conditions, respectively.

**Table 3:** ATP densities in biofilter media. ATP densities reported in ng ATP/g-dry collector; Errors represent standard deviations.

	Experimental Configuration			
Sample depth	Sand/Sat	Sand/Unsat	Biochar/Sat	Biochar/Unsat
Top	9.8 ± 4.8	7.3 ± 1.6	4.1 ± 1.3	4.0 ± 2.1
Middle	3.8 ± 2.5	2.0 ± 0.2	201.3 ± 79.5	240.8 ± 92.1
Bottom	3.1 ± 0.5	2.2 ± 0.5	2.5 ± 1.0	3.9 ± 0.8

ATP concentrations in cells can vary depending on their metabolic condition and stress [52]. Assuming that intracellular concentrations of ATP are on the order of 1 mM [53] and an average cell is shaped as a sphere with a radius of 0.5 μm, there is on the order of 10^−7^ ng ATP per cell.

## Discussion

Regardless of experimental configuration, biofilters consistently attenuated fecal indicator concentrations. This was true for the conditioning phase and during the challenge tests, and for all three fecal indicators tested. Reported log-removals spanned from -0.78 to 2.99, and depended on indicator type, biofilter configuration, and experimental phase with lowest log-removals largely observed for *E*. *coli* at the end of the conditioning phase (after 61 weeks of aging in the field). Further, we found limited leaching of fecal indicators from the biofilters at the end of the experiments.

Biochar amendment of sand biofilters improved the removal of fecal indicators. This was particularly evident at the beginning of the experiments during the first half of the conditioning phase and during the challenge tests at the end of the experiments. Improved removal of fecal indicators in sand biofilters amended with biochar is consistent with results of previous, laboratory-scale studies [11–15,17,18,26,27]. Increased indicator removal in biochar-amended biofilters has been attributed to biochar’s high organic carbon content, which promotes hydrophobic attachment of bacteria to the biochar surface [15]. Furthermore, biochar may also enhance removal relative to sand through its increased surface area, providing additional sites for viral and bacterial attachment [17].

Interestingly, the benefit of biochar amendment for enhancing *E*. *coli* removal diminished over the conditioning phase. After approximately seven months of field conditioning, the difference in *E*. *coli* removal between unamended sand biofilters and biochar-amended sand biofilters was indiscernible. A lack of temporal trends in the influent *E*. *coli* concentrations during conditioning indicates that variation in influent concentration does not explain the gradual decrease in the *E*. *coli* removal capacity of biochar-amended biofilters. We posit that declines in biochar’s performance are attributable in part to biological weathering in the form of biofilm formation. Assuming minimal biofilm was present at the beginning of our experiments, ATP density assays suggest biofilm formation within the biofilters over the experiment’s duration, especially within biochar layers. Assuming physicochemical processes are the main drivers of bacterial removal, biofilm formation in biochar-amended biofilters is likely to reduce *E*. *coli* removal capacity [18,19]. Presence of biofilm influences bacterial removal capacity by altering biochar’s surface roughness and hydrophobicity [18,54,55]. In addition, exposure to NOM in natural stormwater over the experiment’s duration may exhaust biochar’s surface sites and enhance electrostatic repulsion between collectors and cell surfaces following NOM absorption [17,18,21,56].

Surprisingly, the performance of biochar-amended biofilters rebounded during the challenge tests conducted immediately after the conditioning phase. Recall that conditioning was conducted using natural, unamended stormwater while the challenge tests used natural stormwater augmented with a small volume of raw wastewater (1% v/v). We are uncertain as to precisely why the performance of biochar-amended biofilters increased during the challenge tests, but here we outline a number of potential explanations. First, differences in influent chemistry such as ionic strength, pH, and NOM abundance, may have modified *E*. *coli* removal [17,18,21,57,58]. Second, *E*. *coli* indigenous to the natural stormwater may exhibit different transport behavior than those in wastewater. Various *E*. *coli* isolates have demonstrated substantially different transport in simplified porous media experiments at the laboratory scale, owing to differences in their physicochemical properties [14,15,59]. There are substantial differences in the genetic diversity of *E*. *coli* in wastewater and stormwater, which suggests phenotypic diversity that may affect transport [60]. Moreover, we suspect that there may be a difference in the particle-association of wastewater *E*. *coli* and the *E*. *coli* indigenous to stormwater. Limited work has examined the particle association of *E*. *coli* in stormwater [61–63]; results suggest stormwater *E*. *coli* can be both associated with particles and in the free-phase. Wastewater *E*. *coli* may be aggregated in flocs or associated with larger particles. Differences in particle association would affect the removal mechanisms of *E*. *coli*, allowing straining or altering the overall surface properties of *E*. *coli* particles to enhance hydrophobic or electrostatic attachment to geomedia. It is essential for future work to investigate the extent to which fecal indicators are particle-associated in stormwater and how this affects their removal in biofilters. Such work would also inform biofilter design and methods for evaluating biofilter performance in the laboratory and field.

The presence of a saturated zone in biofilters did not significantly impact removal of *E*. *coli* during the conditioning phase. However, a saturated zone was found to enhance removal of all tested fecal indicators during challenge tests. A saturated zone in our system may introduce a “dilution effect” wherein a portion of a biofilter’s effluent consists of water previously retained from a prior wetting event. This volume may exhibit reduced fecal indicator concentrations as additional retention time may promote increased adsorption and die-off [53]. Additionally, a saturated zone may reduce the potential remobilization of fecal indicators [64]. Afrooz and Boehm [19] previously reported that a saturated zone did not influence bacterial removal in biochar-amended biofilters dosed with natural stormwater over 140 days. Given that inclusion of a saturated zone in bioretention systems is important to promote nutrient removal and the fact that a saturated zone improved fecal indicator removal in our challenge tests, it seems prudent to incorporate saturated zones if possible in biofilter design [19,65]. Maintaining a saturated zone at the field scale when there are long periods of dry conditions between storms, however, may prove challenging.

To our knowledge, this study represents the first effort to directly compare enterococci removal in biochar-amended sand biofilters with removal in sand biofilters. Across experimental configuration, biofilters demonstrated greater removal of *E*. *coli* than enterococci, though this difference is less pronounced than that observed previously [19]. The distinct transport of *E*. *coli* and enterococci is attributable to their unique physicochemical properties, including cell membrane composition, motility, shape, surface charge, and hydrophobicity [66–69].

Only one other study has examined the removal of F+ coliphage in a biochar-amended sand biofilter [28], though the work was limited to a laboratory setting. This previous study found that biochar-amendment enhanced F+ coliphage MS2 removal and measured similar removal of MS2 and *E*. *coli* [28]. In the present study, biofilters demonstrated greater removal of F+ coliphage than *E*. *coli*, consistent with the work of Li et al. who studied microbial contaminant removal in conventional biofilter geomedia [33]. F+ coliphage are more similar to human enteric viruses with regards to their fate and transport than bacteria like *E*. *coli* and enterococci [70]. The results of our study suggest that biochar amendment may be a viable strategy for mitigating the public health risks associated with viruses in stormwater runoff.

Mobilization test results indicate minimal mobilization of previously retained fecal indicators. Although concentrations in the effluent were on the order of ambient water quality standards for *E*. *coli* and enterococci (~10^2^ MPN/100 mL), it is inappropriate to expect these concentrations in the effluent of biofilters unless influent concentrations are high as they were purposely designed to be in our challenge tests. While differences in mobilization were observed amongst different experimental configurations, neither geomedia selection nor presence of a saturated zone demonstrated a consistent impact. This contrasts the findings of previous laboratory-scale biofilter studies, which found biochar-amendment to reduce the remobilization of *E*. *coli* under intermittent flows [17,27]. However, those prior studies were conducted in laboratory settings without conditioning with natural stormwater. In the present study, it is possible that biofilm formation or attached NOM led sand and biochar to respond similarly to a wetting front and thus exhibit similar release of attached fecal indicators. Regardless, the limited remobilization is promising and suggested that fecal indicators are sequestered permanently.

## Conclusions

Stormwater biofilters are distributed green infrastructure systems used to mimic natural treatment processes across the urban landscape. Such systems can provide numerous benefits when deployed in a community, including pollutant removal, reduction of runoff flow and volume, urban habitat for plants and animals, and aesthetic benefits [3]. Our study indicates that such systems are capable of removing fecal indicators, including viruses, from natural stormwater runoff.

Fecal indicator removal in biofilters after field conditioning was greater in biochar-amended sand biofilters than sand biofilters by ~0.3 log units and the greatest log-removal for fecal indicator bacteria was approximately 0.8 log units. According to Wolfand et al., such incremental improvements in fecal indicator removal substantially reduce the number of biofilters required to achieve water quality objectives at the watershed scale [71]. However, efforts to further improve removal capacity and sustain the removals observed at the beginning of our conditioning phase (2–3 log units) would be beneficial. Maintaining the high removal performance of biochar may require strategies to promote the in-situ regeneration of geomedia by removing biofilms or attached NOM.

While biochar amendment results in greater microbial removal relative to sand-only biofilters, a decrease in biochar’s microbial removal capacity over the conditioning period could be a concern for field implementation of biochar-amended stormwater biofilters. Though the hydraulic performance of biochar-amended biofilters was not impacted by biological aging over the conditioning period (as supported by the tracer test results), increasingly greater concentrations of active biomass in the biochar layers may result in poor hydraulic performance (i.e., clogging) during long-term operation of biochar-amended biofilters [72,73]. The biochar in our system did not appear to physically degrade. Further investigation is needed to estimate optimum design life of biochar geomedia for stormwater treatment.

The study described herein aimed to assess the performance of biochar-amended biofilters under simulated field conditions. Biofilters were aged outdoors and subject to periodic wetting with natural stormwater bearing typical fecal indicator concentrations and fecal indicators that were not laboratory-cultured. These efforts represent a substantial improvement in mimicking realistic conditions over what has been achieved by previous laboratory studies. However, the biofilters assessed here remained small relative to those typically implemented in the field. For example, in Los Angeles County, biofilters are typically on the order of 1 m deep [74] and storms may route larger volumes to the biofilters than those tested in this study. Future work should assess performance of biochar-amended biofilters in the field with respect to improving stormwater quality.

## Supporting information

S1 Fig Influent and effluent *E. coli* concentrations from conditioning phase. *E. coli* concentrations outside the limits of quantification are omitted. Influent concentrations from dates where effluent was not sampled are omitted for clarity. Each point reflects the median *E. coli* concentrations determined via Monte Carlo simulation while error bars represent the interquartile range of concentrations generated via the simulation.
(TIFF)

S2 Fig Br- breakthrough curves from final tracer test. Each point represents the average normalized Br^-^ concentration from duplicate technical replicates and triplicate biological replicates for each experimental condition. Error bars represent 1 standard deviation. Note that the final tracer test was conducted without pre-saturating the biofilter media.
(TIF)

S1 Table Details of biochar pyrolysis temperature. (PNG)

S2 Table Conditioning phase effluent *E. coli* concentrations. (PNG)
