# Supplementary figures and images for: Fecal indicator bacteria and virus removal in stormwater biofilters: Effects of biochar, media saturation, and field conditioning
Source: PLoS One. 2019 Sep 25;14(9):e0222719. doi: 10.1371/journal.pone.0222719 (PMC6760807; doi:10.1371/journal.pone.0222719)

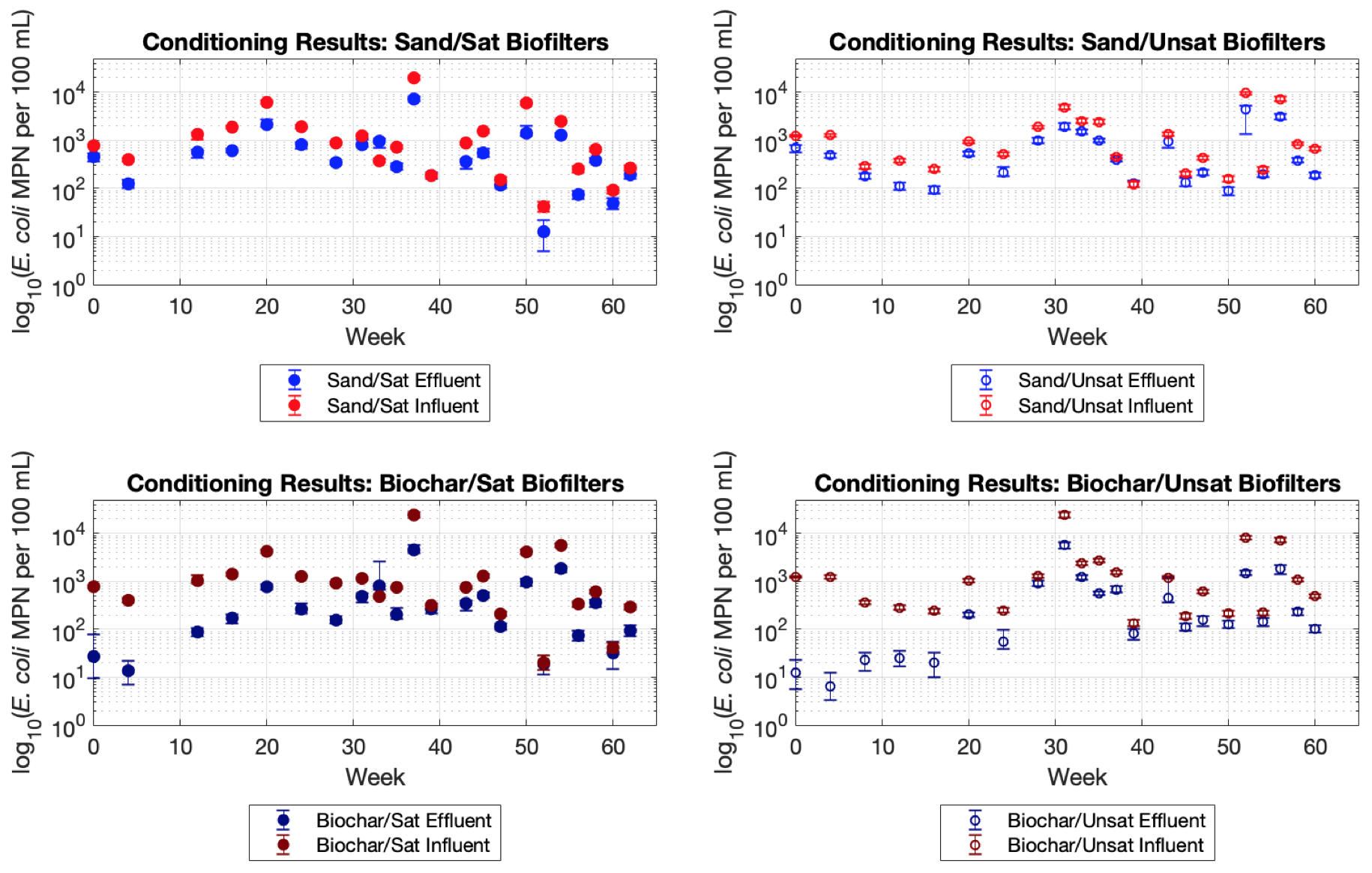

Supplement: S1 Fig — E. coli concentrations outside the limits of quantification are omitted. Influent concentrations from dates where effluent was not sampled are omitted for clarity. Each point reflects the median E. coli concentrations determined via Monte Carlo simulation while error bars represent the interquartile range of concentrations generated via the simulation. (TIFF) [file pone.0222719.s001.tiff]

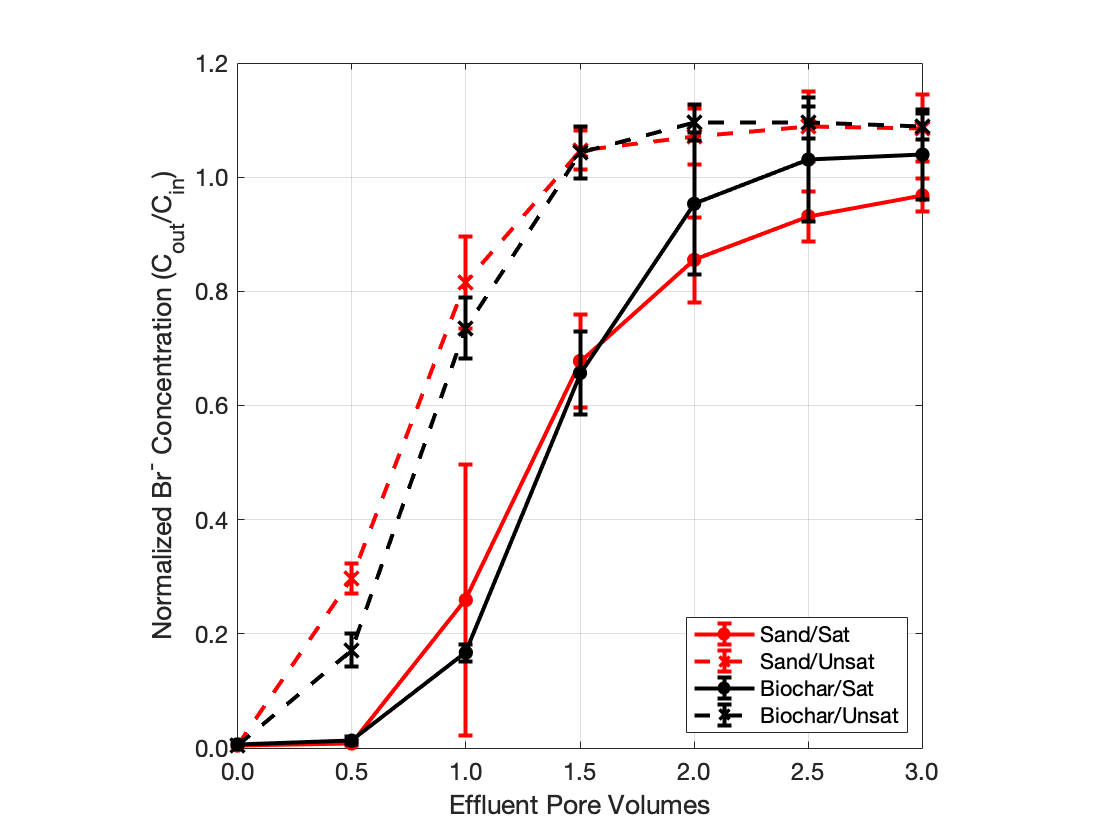

Supplement: S2 Fig — Each point represents the average normalized Br- concentration from duplicate technical replicates and triplicate biological replicates for each experimental condition. Error bars represent 1 standard deviation. Note that the final tracer test was conducted without pre-saturating the biofilter media. (TIF) [file pone.0222719.s002.tif]

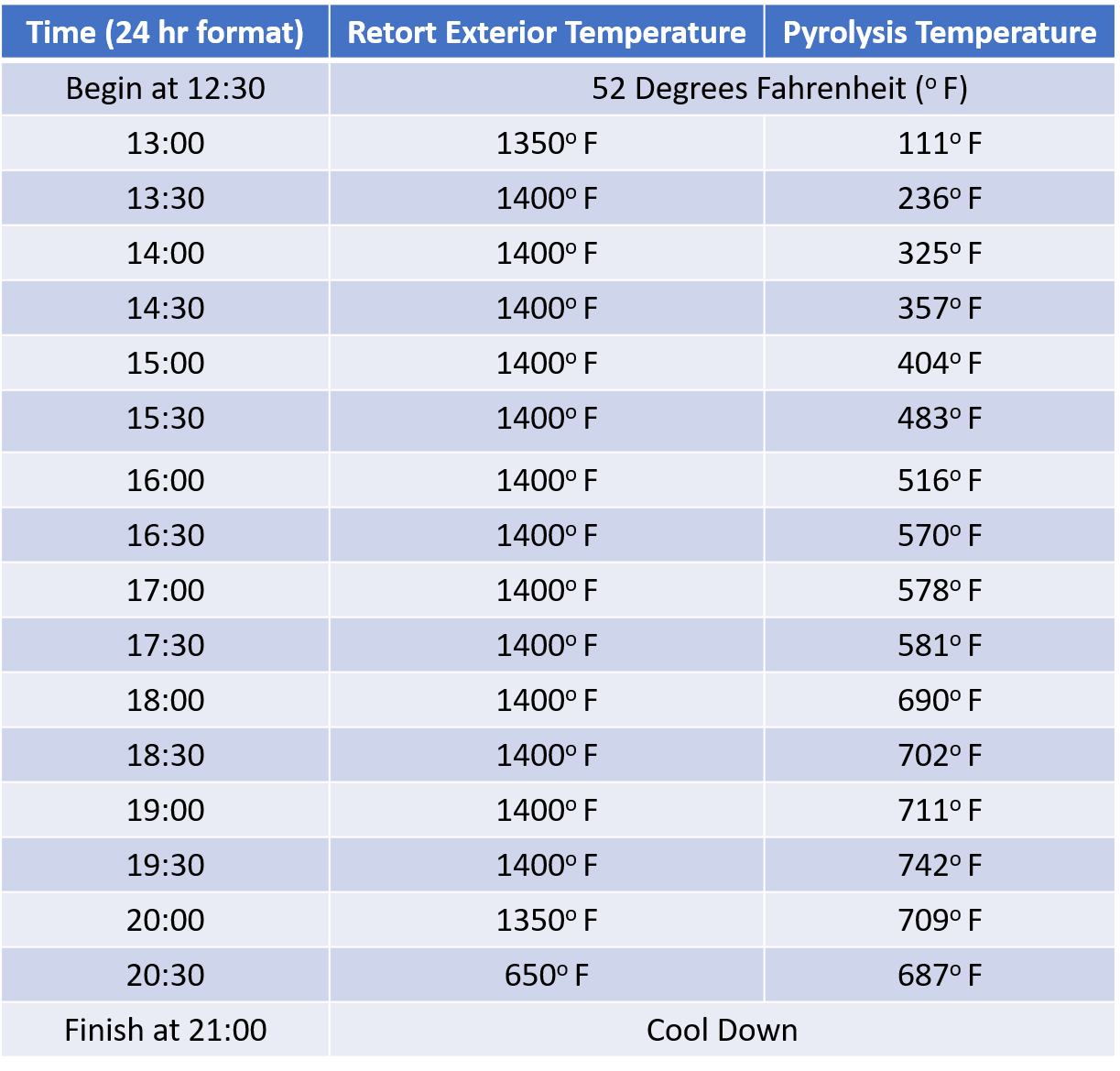

Supplement: S1 Table — (PNG) [file pone.0222719.s003.png]

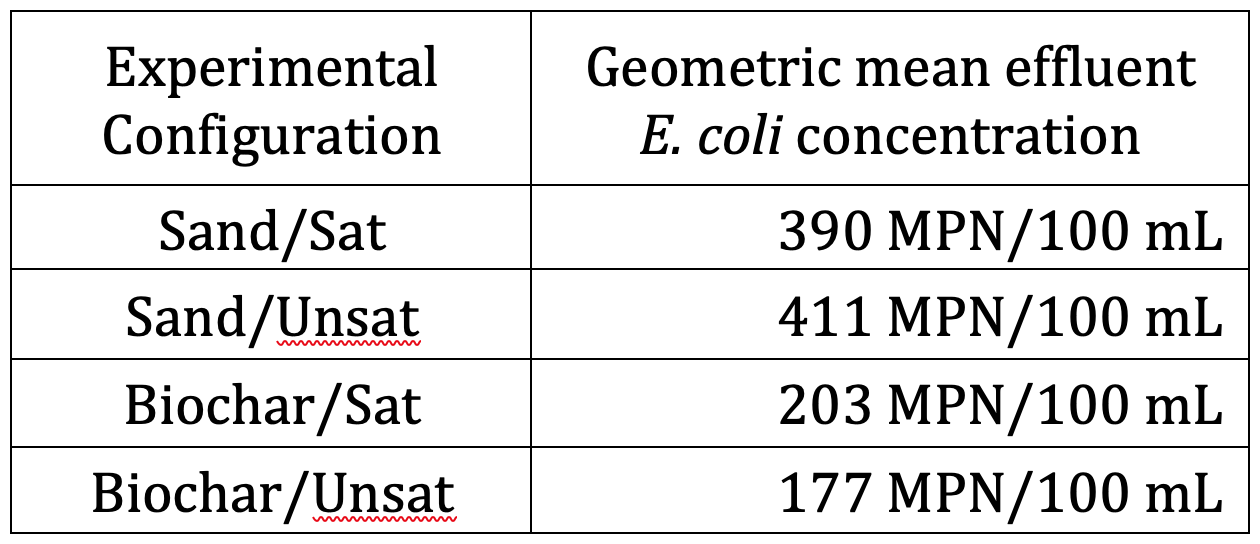

Supplement: S2 Table — (PNG) [file pone.0222719.s004.png]
